# Neutrophil-to-Lymphocyte Ratio (NLR) in NSCLC, Gastrointestinal, and Other Solid Tumors: Immunotherapy and Beyond

**DOI:** 10.3390/biom13121803

**Published:** 2023-12-18

**Authors:** Mirta Mosca, Maria Concetta Nigro, Rachele Pagani, Andrea De Giglio, Alessandro Di Federico

**Affiliations:** 1 Department of Medical and Surgical Sciences, S. Orsola-Malpighi University Hospital, University of Bologna, 40138 Bologna, Italy; mirta.mosca@studio.unibo.it (M.M.); mariaconcetta.nigro@studio.unibo.it (M.C.N.); rachele.pagani@studio.unibo.it (R.P.); alessandr.difederic2@unibo.it (A.D.F.); 2 Medical Oncology, IRCCS Azienda Ospedaliero-Universitaria di Bologna, 40138 Bologna, Italy

**Keywords:** NLR, neutrophil-to-lymphocyte ratio, immunotherapy, prognosis

## Abstract

In the era of immunotherapy, identifying biomarkers of immune system activation has become a high-priority challenge. The blood neutrophil-to-lymphocyte ratio (NLR) has been largely investigated as a biomarker in several cancer types. NLR values have been shown to mirror the tumor-induced inflammatory status and have been demonstrated to be a reliable prognostic tool across stages of disease and therapeutic approaches. When integrated with other biomarkers of response to immunotherapy, such as PD-L1, tumor mutational burden, and tumor-associated immune cells, the NLR may allow to further stratify patients with different likelihoods of deriving a significant clinical benefit. However, despite its accessibility, low cost, and easy interpretation, the NLR is still poorly used as a prognostic tool in daily clinical practice. In this review, we analyze the role of the NLR in defining the relationship between cancer and the immune system, its usefulness in daily clinical practice, and its relationship with other established or emerging biomarkers of immunotherapy outcomes.

## 1. Introduction

The interplay between cancer and the immune system is highly complex and essential for many steps of cancer development, growth, and spread from the organ of origin through the organism. While the main role of immune cells towards cancer is to prevent cancer formation by the timely recognition and elimination of transformed cells, the inflammatory response has been proven to underlie many processes that ultimately promote cancer growth and progression [1,2,3]. In recent times, advances in immunology and immuno-oncology led to the development of a new class of anticancer agents that take advantage of the innate ability of the immune system to kill cancer cells, generically called immunotherapies. By targeting the key immune checkpoints involved in the relationship between cancer and the immune system, including the programmed cell death receptor 1 (PD-1), its ligand (PD-L1), and cytotoxic T lymphocyte antigen 4 (CTLA-4), these drugs prevent immunosuppressive interactions that lead to immune escape and restore the killing activity of immune cells towards tumor cells [4]. The so-called immune checkpoint inhibitors (ICI) rapidly became part of the standard of care treatment algorithm of the majority of cancer types in the advance setting and, more recently, in the adjuvant and neoadjuvant settings. However, biomarkers of the efficacy of immunotherapies are still lacking for many tumors, and there is large room for improvement for other tumor types where some biomarkers have shown a predictive value, such as PD-L1 expression and TMB in patients with non-small cell lung cancer (NSCLC) [5]. The expression of PD-L1, for instance, guides the choice of the first-line therapeutic options for patients with non-oncogene-addicted NSCLC, but its reliability in distinguishing patients who will derive a significant benefit from immunotherapy from those who will not is far from being perfect [6]. In this context, identifying biomarkers of inflammation and immune system activation that may serve as predictors of immunotherapy outcomes has become a high-priority challenge [7,8,9]. Several scores taking into account easily detectable inflammatory markers have been correlated with the survival of patients with solid tumors, showing to have at least prognostic value. One example is the Glasgow Prognostic Score (GPS), which considers the increase in the serum C-reactive protein and the decrease in serum albumin concentration, two events associated with the acute inflammatory phase [10,11]. A high GPS score generally correlates with worse survival outcomes in patients with solid tumors [10]. Inflammatory cytokines, lactate dehydrogenase, as well as the presence and density of specific immune cell subsets represent additional biomarkers mirroring the systemic inflammatory status [3,12,13]. In particular, the predominance of neutrophils in association with low lymphocyte levels is typical of the non-specific acute inflammatory response. Therefore, the neutrophil-to-lymphocyte ratio (NLR) has been largely studied with the hypothesis of being a potentially reliable predictive or, at least, prognostic factor across different cancer types [14,15,16]. The NLR can be easily calculated from routine blood tests, potentially serving as an accessible and low-cost tool to help evaluate the prognosis of patients with cancer. Despite its accessibility, low cost, and easy interpretation, the NLR is still poorly used as a prognostic tool in daily clinical practice.

In this review, we provide a comprehensive and up-to-date overview on the NLR, its ability to serve as a mirror of the status of the immune system in patients with solid tumors, and its role as a predictor of outcomes across cancer types, the setting of diseases, and treatment strategies. Moreover, we discuss the usefulness of the NLR in daily clinical practice, summarizing the evidence regarding its possible integration with other established or emerging biomarkers of outcomes to immunotherapies to enhance their accuracy in stratifying the likelihood of deriving a significant clinical benefit from these treatments.

## 2. NLR in the Relationship between Cancer and the Host Immune System

Alterations in local and systemic inflammation, myelopoiesis, and leukocyte count are considered hallmarks of cancer evolution [17,18]. The correlation between systemic inflammatory response and tumor behavior fostered the investigation of the NLR as a possible prognostic and predictive biomarker for patients receiving multiple treatment strategies, including chemotherapy and immunotherapy [19,20,21,22]. Many studies showed a deep connection between NLR values and the activation of the innate immune response, suggesting that the NLR might mirror the tumor-induced inflammatory status. In fact, a high NLR was found to be correlated with increased peritumoral macrophage infiltrate and high levels of several pro-inflammatory cytokines, including IL-1ra, IL-6, IL-7, IL-8, IL-12, IL-17, MCP-1, and PDGF-BB [23,24,25,26,27,28,29,30,31,32,33,34,35,36,37,38]. High NLR values also showed to be associated with a high concentration of tumor-associated neutrophils (TANs), which may play a dual role in favoring or contrasting tumor development under different stimuli coming from cancer and immune cells [39,40]. TANs are a heterogeneous population recruited and then polarized from the pool of circulating neutrophils. They can be subdivided into N1 neutrophils, that mainly elicit anti-tumor activities by activating innate and adaptive immune cells, and N2 neutrophils, that mainly favor tumor growth through the induction of neo-angiogenesis and stroma remodeling [41]. In addition, the tumor genomic context has been shown to be associated with neutrophil infiltration through crosstalk modulation. The loss of *TP53* in a *KRAS* G12D-mutated pancreatic cancer murine model modulated cytokines such as CXCL1 and CXCL and determined the enhanced infiltration of CD11b+ myeloid cells, including monocytes, macrophages, and neutrophils [42,43].

Several studies reported that, in healthy conditions, circulating and tissue neutrophils presented high phenotypic and functional heterogeneity, which were regulated by maturation and aging levels and by the tissue microenvironment. Circadian oscillation and aging influenced the type of chemokine receptors expressed by neutrophils, their pattern recognition receptors, their inflammasome, and their production of extracellular traps, such as their capacity to migrate [41]. In normal conditions, to limit their potential damage, circulating neutrophils do not have the capacity to produce extracellular traps before they penetrate tissues [41].

In cancer patients, neutrophil differentiation and the maturation process seem to be profoundly altered. In fact, in murine cancer models, neutrophils presented a transcriptional program that made them able to produce reactive oxygen species (ROS), nitric oxide, and arginase 2 with a potential inhibition on T-cell proliferation [42]. According to their polarization in a pro-tumor sense, neutrophils also presented a high expression of ARG1, CCL17, and CXCL14, and a low expression of CXCL10, CXCL13, CCL6, and TNF.

The ROS produced by neutrophils were associated with damage and genetic instability in epithelial cells, and the DNA damage was also elicited through the production of microRNAs, that promoted DNA double-strand breaks in epithelial cells [42].

Moreover, in murine models, neutrophil extracellular traps-associated molecules such as high mobility protein B1, neutrophil elastase (NE), and metalloproteinase 9 (MMP9) could stimulate the proliferation of cancer cells and the formation of metastases through the entrapment of circulating tumor cells [42].

**Figure 1:**
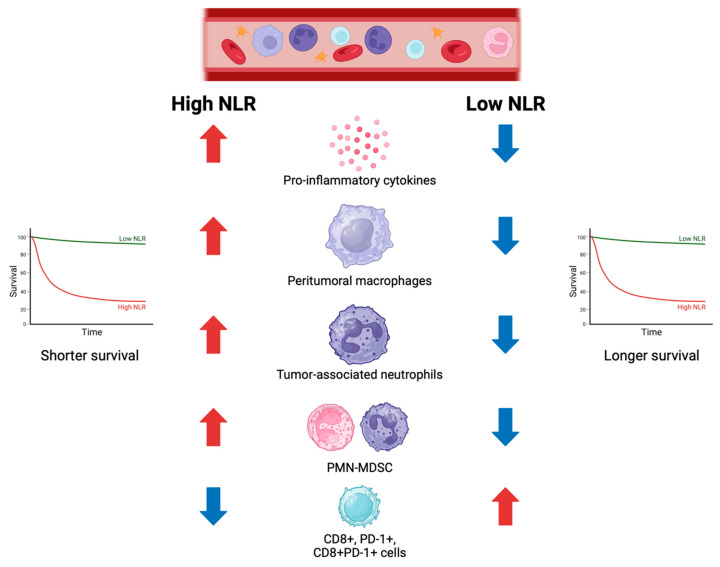
Association between neutrophile-to-lymphocyte ratio (NLR), immune cells, and survival outcomes.

Higher levels of TAN were found to be associated with higher serum IL-8 levels and worse survival and ICI efficacy in two studies including patients with advanced NSCLC, melanoma, urothelial carcinoma, and renal cell carcinoma [44,45]. Interestingly, antagonizing neutrophils with a CXCR1 and CXCR2 inhibitor improved the sensitivity to PD-1 inhibition in a preclinical model of lung carcinoma [46]. Circulating neutrophils can also be categorized based on their cellular density into low- or high-density neutrophils following the Ficoll-Hypaque centrifugation [47]. High-density neutrophils demonstrated anti-tumor functions, while low-density neutrophils have mainly immunosuppressive activity and have been consistently associated with high NLR and poor prognosis in patients with cancer [47,48]. Myeloid-derived suppressor cells (MDSC) are immature myeloid cells recruited from the bone marrow by tumor-derived factors, such as the vascular endothelial growth factor (VEGF) and granulocyte-macrophage colony-stimulating factors (GM-CSF). In humans, MDSC can be divided into monocytic MDSC (M MDSC), polymorphonuclear MDSC (PMN MDSC), which also comprise immature neutrophils, and early MDSC [49]. In humans, granulocytic MDSC are represented by CD66b+ CD14− CD11b+ CD15+ cells, while monocytic MDSCs are represented by CD11b+ CD14+ HLA-DR^low^ CD15− cells [50]. Several studies suggested that the tumor can enhance myelopoiesis in the bone marrow and in extramedullary organs by producing the granulocyte colony-stimulating factor, which acts by expanding granulocyte myelocyte progenitor cells and other early neutrophil progenitors’ clones [51,52]. When accumulated in the tumor microenvironment, MDSCs produce and release IL-10, TGF-β, and other molecules that promote neo-angiogenesis and exert immunosuppressive activity, facilitating tumor growth, survival, and progression [41,47,48]. NLR and PMN MDSC values are strongly correlated with each other, as PMN MDSC as a group also comprise neutrophils that are accounted for in the calculation of NLR [53]. Consistently with their biologic function, high levels of PMN MDSC and low-density neutrophils have been associated with a more advanced stage of disease and a poorer cancer prognosis [47,48,53,54,55]. Neutrophils have been shown to exert a direct procarcinogenic effect by producing reactive oxygen species that amplify the DNA damage initiated by the exposure to a carcinogen, and to facilitate the interaction between melanoma cells and the endothelium to promote cancer spreading through blood vessels [56,57]. While in the bloodstream, the presence of clusters of cancer cells and neutrophils has shown a higher metastasizing potential compared to cases where cancer cells do not form clusters with neutrophils [58]. Moreover, even before metastasizing, cancer-induced neutrophil accumulation and extracellular matrix remodeling in distant sites from the primary tumor form favorable premetastatic niches for future cancer spread [59,60]. However, neutrophils can also act by limiting tumor proliferation and killing cancer cells through the production of H2O2 and the interaction with the transient receptor potential cation channel M2 (TRPM2) [61]. Given the complexity of the interactions between the immune system and cancer, the NLR was questioned to be an oversimplifying tool as it only includes neutrophils and lymphocytes in its assessment. Differently from the NLR, the derived NLR (dNLR) indirectly takes into account immune cells other than neutrophils and lymphocytes as it is calculated from neutrophils (ANC) and white blood cell (WBC) counts with the formula dNLR = ANC/(WBC-ANC) [23]. Similarly to the NLR, the dNLR has been associated with poor outcomes by many studies across cancer types and may serve as a surrogate of the density of some type of tumor-associated immune cells [25]. In a retrospective study conducted on 221 patients with advanced NSCLC and a high programmed cell death ligand 1 (PD-L1) tumor proportion score (≥50%) treated with first-line pembrolizumab, patients with low dNLR (<2.6) had significantly increased tumor-associated CD8+, FOXP3+, and PD-1+ immune cells, and PD-1+ CD8+ T cells identified by multiplexed immunofluorescence compared to patients with high dNLR (≥2.6) [62]. This study, among others, established the close connection between the dNLR and specific immune features in the tumor immune microenvironment. The main immune correlates of the NLR and dNLR values are summarized in Figure 1.

## 3. Clinical Applications of NLR and Derived Scores

### 3.1. Prognostic Value under Chemotherapy

NLR is a cost-effective and reliable tool that can be exploited in a wide number of scenarios during daily clinical practice. High NLR values have been associated with features of tumor aggressiveness across many cancer types, including the presence of microvascular invasion and lymph node involvement in early-stage operable cancers or with the presence of multiple distant metastases in advanced tumors [25,26,27]. NLR values have been demonstrated to be reliable predictors of prognosis for patients with different malignancies across stages of disease and therapeutic approaches. In patients with metastatic colorectal cancer, a high NLR (>5) was found to be independently associated with reduced overall survival (OS) [63]. In addition, after one cycle of chemotherapy, patients with high NLR values (>5) who had a decrease in the NLR under the value of 5 achieved a significantly longer progression-free survival (PFS) compared to patients who still had an NLR > 5 [63]. Similarly, a high NLR predicted shorter disease-free survival (DFS) and OS in patients with early-stage gastrointestinal tumors who received neoadjuvant treatment followed by surgery, despite showing no association with a pathological response [27,28,29,30,31,32,33,34,35,36,37]. A higher risk of disease recurrence was observed among patients with hepatocellular carcinoma (HCC), who underwent living-donor liver transplantation and had high pre-transplantation NLR levels [23]. In addition, a study showed an increased mortality risk among patients with localized NSCLC who had undergone complete surgical resection and had a high pre-operative NLR [24]. Consistently, a high NLR was associated with worse outcomes to chemotherapy and other treatment strategies in patients with advanced cancers, including colorectal, prostate, head and neck, and lung [63,64,65]. Variations in the NLR following anti-cancer treatments may also have an important prognostic role in patients with advanced solid tumors, as suggested by a systematic review of studies showing a correlation between the decrease in the NLR after chemotherapy and better outcomes [27].

### 3.2. Prognostic Value under Immunotherapy

As previously described, an immunosuppressive tumor microenvironment characterized by low-density neutrophils, M2 polarized macrophages, and FOXP3+ regulatory T cells may facilitate the progression of tumorigenesis [18,66,67]. Recently, the advent of ICI targeting PD-1 or PD-L1 has drastically changed the outcomes of patients with solid tumors [68]. These monoclonal antibodies prevent inhibitory interactions between immune and cancer cells, restoring the activity of the immune system against the tumor. However, only a minority of patients respond to ICI, and an even lower proportion of them derive durable clinical benefit [69,70,71,72]. Identifying biomarkers able to maximize the accuracy of the selection of patients who will achieve a meaningful benefit is therefore crucial to offer more effective therapies to patients who are not likely to benefit from these agents and, at the same time, to avoid unnecessary potential toxicities to patients who will not benefit from ICI. PD-L1 expression and tumor mutational burden (TMB) are both considered independent predictive biomarkers of response to immunotherapy; however, they still lack complete reliability, as objective and durable responses can be observed among patients with low levels of one or both biomarkers, while both primary and acquired resistance are frequent events, even in the presence of high PD-L1 and/or TMB levels [73,74,75]. The complex and dynamic interaction between cancer and the immune system during ICI-based treatments can hardly be predicted by the evaluation of a single biomarker [76,77,78,79,80,81,82,83]. Instead, integrating together multiple variables, such as PD-L1, TMB, genomic alterations (e.g., *KRAS*, *STK11*, and *KEAP1* molecular status in the case of NSCLC), and inflammatory markers may result in enhanced predictive accuracy. A correlation between high baseline NLR and worse outcomes to IL-2 and nivolumab therapies was initially observed among patients with renal cell carcinoma and NSCLC [84,85]. Many other studies have investigated the potential correlation between the NLR and response to immunotherapy among patients with different malignancies, especially lung cancer and melanoma [20,86,87,88,89,90,91,92]. In a retrospective study that included 221 patients with advanced NSCLC and high PD-L1 expression (≥50%) who received first-line pembrolizumab, low levels of dNLR (<2.6), compared to high levels (≥2.6), were found to be associated with a significantly higher objective response rate (52.4% vs. 24.7%, *p* < 0.001) and longer median PFS (10.4 vs. 3.4 months, *p* < 0.001) and OS (36.6 vs. 9.8 months, *p* < 0.001). The statistical significance of these findings was maintained even after adjusting for potential confounders in a multivariable model, suggesting the role of dNLR as an independent prognostic factor [62]. A consistently independent effect of the NLR on OS in patients with NSCLC, renal cell cancer, and melanoma was observed in a sub-analysis of the INCIDIa-2 study, which showed a significantly longer OS in patients with low NLR levels (<3.4) [93]. Moreover, as already observed with chemotherapy, a dynamic evaluation of the NLR may help with monitoring the efficacy of ICI. An early decrease in the NLR during treatment with anti-PD-(L)1 agents was shown to correlate with an enhanced INF-γ response, improved antitumor activity, and significantly better survival outcomes [81,82]. On the contrary, an early increase in the NLR after two cycles of treatment with nivolumab in patients with NSCLC was associated with significantly shorter PFS [83]. Similar results were observed in patients with renal cell carcinoma treated with ICI, as the reduction in the NLR after 6 ± 2 weeks from ICI initiation compared to baseline levels was significantly associated with improved outcomes [84,85]. Some studies have also tried to improve the predictive accuracy of the NLR by combining it with other easily detectable markers, therefore without altering accessibility complexity compared to the evaluation of the NLR as a single biomarker. An example is the Lung Immune Prognostic Index (LIPI), which is a simple risk-stratification score based on LDH levels and dNLR [94]. LDH is the enzyme responsible for transforming pyruvate to lactate, and its levels increase in the case of high cellular metabolic activity and turnover. For these reasons, LDH can be often found elevated in patients with aggressive solid tumors, such as melanoma, and hematological malignancies, and high levels have been associated with poor prognosis in several tumors, including melanoma and renal carcinoma [95,96,97]. The retrospective study by Mezquita et al. was the first to evaluate the utility of the LIPI score in clinical practice [94]. According to the LIPI score, 466 patients with advanced NSCLC treated with PD-1/PD-L1 inhibitors or standard chemotherapy were classified into three prognostic groups (good, intermediate, and poor) based on LDH and dNLR levels. Compared to patients in the good prognostic group, those in the intermediate and poor prognostic groups had significantly shorter median PFS and OS when treated with immunotherapy, but no difference was observed among patients receiving chemotherapy [94]. Subsequent studies have confirmed the reliability of the LIPI score in stratifying the prognosis of patients treated with immune-checkpoint inhibitors in several other cancer types, including melanoma, renal cancer, small cell lung carcinoma, head and neck squamous cell carcinoma, urothelial cancer, and triple-negative breast cancer [98,99,100,101,102,103,104,105].

## 4. Relationship of NLR with Other Biomarkers

### 4.1. PD-L1 Expression and Tumor-Infiltrating Lymphocytes

The role of PD-L1 expression in predicting outcomes to ICI remains controversial in several advanced malignancies, as discordant results have been frequently reported [106,107,108]. Even in cancer types where PD-L1 has a key role in the decisional treatment algorithm, such as NSCLC, it is considered an imperfect biomarker since tumor responses occur even in patients with low or absent PD-L1 expression, and poor outcomes are not uncommon even in patients with high PD-L1 expression of ≥50%. Besides PD-L1, several other biomarkers have been shown to predict ICI efficacy, such as the density of tumor-infiltrating lymphocytes (TILs) and TMB [109,110,111,112,113,114]. Different studies showed that combining the NLR with other biomarkers might enhance their accuracy in identifying patients who derive a significant benefit from immunotherapies as well as the general prognostic value of NLR when evaluated alone (Table 1).

Because high NLR and low TILs density, when evaluated as single biomarkers, were significantly correlated with survival among patients with laryngeal cancer, the relationship between NLR, TILs, and PD-L1 expression and their impact in predicting disease-free survival (DFS) was investigated in patients with this tumor [106]. Patients with a PD-L1 combined proportion score (CPS) ≥1% and a TILs count rate ≥30%, both of which were associated with a lower median NLR, achieved a significantly prolonged DFS compared to patients with negative PD-L1 CPS or a TILs count rate <30% [106,115]. Specific subpopulations of lymphocytes might be associated with blood NLR levels, as suggested by a study conducted in 288 patients undergoing curative surgery for gastric cancer [116]. CD4^+^ immune cell density was significantly higher among patients with a low NLR; on the contrary, CD3^+^ or CD8^+^ immune cell densities did not show an association with the NLR [116]. The ATTRACTION-2 was a randomized phase three clinical trial that showed a survival benefit with nivolumab versus a placebo in patients with advanced gastric cancer that was refractory to two or more lines of chemotherapy [117]. Although the primary endpoint of OS was met, the ORR to nivolumab was as low as 11.2%, highlighting the need for predictive biomarkers of immunotherapy benefit. In a post hoc analysis of the same trial, patients treated with nivolumab, as compared to those who received a placebo, seemed to show numerically improved PFS in the case of the positive tumor PD-L1 CPS (≥1%) and a low NLR at baseline, while no improvement was observed in patients with negative PD-L1 CPS (<1%) and a high baseline NLR [118]. Similarly, in another study, the NLR was proved to increase the accuracy of other biomarkers, including PD-L1 CPS and TILs density, in predicting the PFS and OS of patients with locally advanced gastric cancer treated with neoadjuvant chemotherapy, highlighting the importance of a combined biomarker evaluation [119]. High NLR values were found to correlate with low TILs also in patients with colorectal cancer, and a combined evaluation of these two biomarkers improved the prediction of survival compared to TILs alone in patients with resected stage III colorectal cancer who received adjuvant FOLFOX chemotherapy [120,121]. The retrospective study by Gawinski et al. analyzed 50 patients with locally advanced left-sided colorectal cancer who underwent surgery with radical intent, dividing them into groups according to high or low NLR, platelet-to-lymphocyte ratio, and lymphocyte-to-monocyte ratio [122]. The study assessed the density of CD3^+^ and CD8^+^ lymphocytes in resected specimens in the center of the tumor and in the invasive margin, finding that the levels of CD3^+^ lymphocytes in the center of the tumor were significantly higher in patients with low pre-treatment NLR than in patients with high pre-treatment NLR. The five-year OS rate was significantly higher in patients with a high lymphocyte-to-monocyte ratio value compared to that of patients with a low lymphocyte-to-monocyte ratio; a similar difference was observed in patients with a low NLR value, which had a better OS than patients with a high NLR. No statistically significant difference in terms of OS was detected in the low and high platelet-to-lymphocyte ratio group [122]. The prognostic impact of tumoral PD-L1 expression in relationship with NLR in patients with resected stage I lung squamous cell carcinoma was evaluated by Tashima et al. [108]. A positive PD-L1 expression in tumor cells (≥1%) was significantly associated with shorter survival only in patients with low NLR, while it was not found to have an impact on survival among patients with high NLR; moreover, having a low NLR and a negative PD-L1 expression was independently associated with improved recurrence-free survival and OS in a multivariate model [108]. These results have been replicated in a cohort of patients with resected stage I–III NSCLC, in which the population having high NLR and positive PD-L1 expression (≥1%) achieved significantly shorter DSF and OS compared to the rest of the cohort; these findings were further confirmed in a study conducted by Xia et al., which reported consistent results [112,123]. In patients with previously treated advanced NSCLC, the POPLAR and OAK randomized clinical trials established the superiority of atezolizumab, a PD-L1 inhibitor, over standard chemotherapy with docetaxel, leading to the approval of the PD-L1 inhibitor as a second-line treatment option after progression to first-line platinum-based chemotherapy, regardless of PD-L1 expression levels [124,125]. A post hoc analysis of these two studies was focused on the role of NLR as an additional biomarker other than PD-L1 expression [126]. The study found that the combination of the NLR and PD-L1 expression was more accurate in predicting the outcomes to atezolizumab compared to the single evaluation of PD-L1 expression. Patients with both a high NLR and negative PD-L1 expression (<1%) treated with atezolizumab had a significantly shorter OS than patients with both a low NLR and positive PD-L1 (≥1%) or than patients with NLR low/PD-L1 negative or NLR high/PD-L1 positive status, who showed intermediate outcomes [126].

**Table 1:** Main studies conducted on patients with NSCLC or gastrointestinal neoplasms, investigating the association between neutrophils-to-lymphocyte ratio (NLR), other biomarkers, and immune-checkpoint inhibitors (ICI) efficacy.

First Author	Cancer Type	Setting	Treatment	Combined Biomarker	Effect on Outcomes
Alessi et al. [62]	NSCLC	Advanced, first-line	Pembrolizumab	PD-L1 (population with PD-L1 TPS ≥ 50%)	ORR, PFS, and OS improvement in the low-NLR group
Cortellini et al. [126]	NSCLC	Advanced, second-line	Atezolizumab	PD-L1 (TPS< vs. ≥1%)	PFS and OS improvement with low-NLR/positive-PD-L1
Kao et al. [127]	NSCLC	Advanced, first- or subsequent- line	Immune checkpoint inhibitor	TMB (< vs. ≥10 mut/Mb)	OS improvement with low-NLR/high-TMB
Xia et al. [123]	NSCLC	Advanced, any line, with brain metastasis	Chemotherapy or EGFR-TKI	PD-L1 (TPS< vs. ≥1%)	OS improvement with low-NLR/negative-PD-L1
Tashima et al. [108]	NSCLC	Resected, stage I	Surgery only	PD-L1 (TPS< vs. ≥1%)	RFS and OS improvement with low-NLR/negative-PD-L1
Wang et al. [112]	NSCLC	Resected, stage I–III	Surgery only or followed by adjuvant therapy	PD-L1 (TPS< vs. ≥1%)	DFS and OS improvement with low-NLR/negative-PD-L1
Kim et al. [118]	Gastric cancer	Advanced, third- or later line	Nivolumab	Serum Na (< vs. ≥135 mmol/L)	ORR and PFS improvement with low-NLR/high-Na
Zurlo et al. [119]	Gastric cancer	Locally advanced	Chemotherapy	TILs (< vs. ≥0.2) and PD-L1 CPS (< vs. ≥1%)	PFS and OS improvement with low-NLR/low-TILs/high-PD-L1
Cha et al. [121]	Colorectal cancer	Resected stage III, adjuvant	Chemotherapy	TILs (score)	OS improvement with low-NLR/high-TILs
Valero et al. [128]	Pan-cancer	Advanced, any line	Immune checkpoint inhibitor	TMB (< vs. ≥20th percentile in each tumor type)	ORR, PFS, and OS improvement with low-NLR/high-TMB

NSCLC: non-small cell lung cancer; PD-L1: programmed death receptor ligand 1; TPS: tumor proportion score; CPS: combined proportion score; NLR: neutrophil-to-lymphocyte ratio; TILs: tumor-infiltrating lymphocytes; ORR: objective response rate; RFS: relapse-free survival; DFS: disease-free survival; PFS: progression-free survival; OS: overall survival; EGFR-TKI: epidermal growth factor receptor tyrosine kinase inhibitor.

### 4.2. Tumor Mutational Burden

As the TMB has been demonstrated to be a reliable predictive factor of ICI efficacy in patients with various tumors, including NSCLC, some studies investigated whether combining its evaluation with that of NLR may improve the predictive accuracy of these two biomarkers when assessed separately. The study by Valero et al. analyzed a retrospective cohort of 1714 patients with 16 different types of cancer to investigate the value of combining NLR and TMB to predict tumor response and patients’ survival to ICI [128]. This study showed that combining the two biomarkers can stratify patients that benefit from ICI more accurately compared to when they were evaluated separately [128]. Patients with a low NLR and high TMB showed the highest ORR (38.3%) and the longest median PFS and median OS to ICI. Conversely, patients with a high NLR and low TMB showed the lowest ORR (16.8%) and the shortest median PFS and OS, while intermediate outcomes were observed in those with a high NLR and high TMB or low NLR and low TMB. In a multivariable analysis, patients with a low NLR and high TMB had a more than three-fold enhanced likelihood of deriving clinical benefits from ICI compared to patients with a high NLR and low TMB (odds ratio 3.48, *p* < 0.0001). Importantly, these findings were confirmed in a recent retrospective study that enrolled patients with advanced NSCLC treated with ICI, which showed that combining TMB, PD-L1 expression, and NLR improves the accuracy of predicting the ORR, time to disease progression, and OS compared to the TMB when evaluated alone, highlighting again the importance of a combined biomarker approach [127]. Prospective trials are necessary to evaluate the predictive worth of the NLR in the effectiveness of immunotherapy and its association with tumor load, systemic inflammation, and the tumor microenvironment, given their critical prognostic significance.

## 5. Conclusions

The NLR was demonstrated to have a strong prognostic value in patients with solid tumor across cancer types, stages of disease, and treatment strategies. However, the close relationship between the NLR and specific immune markers reflecting immune system activation might suggest that the NLR is even more accurate in predicting survival among patients treated with immunotherapies. When evaluating other biomarkers of response to ICI, such as PD-L1 expression, TMB, and tumor-associated immune cells (e.g., TILs density), combining the assessment of the NLR may allow to further stratify patients with different likelihoods of deriving a significant clinical benefit while not increasing costs nor adding complexity to the evaluation. It is noteworthy that the evidence substantiating the predictive capability of NLR is rather low and predominantly rests on retrospective or post hoc analyses. Therefore, given the high accessibility of the NLR and the consistent results showing its reliability in predicting the prognosis of patients with different cancer types reported in the literature, the evaluation of the NLR or its validated derived measures, such as the dNLR and LIPI score, should serve as a prognostic tool in daily clinical practice.
